# Low-glucose culture environment can enhance the wound healing capability of diabetic adipose-derived stem cells

**DOI:** 10.1186/s13287-023-03478-2

**Published:** 2023-09-04

**Authors:** Chun-Wei Li, Tai-Horng Young, Mu-Hui Wang, Ming-Ying Pei, Tsung-Yu Hsieh, Chia-Lang Hsu, Nai-Chen Cheng

**Affiliations:** 1 Department of Plastic and Reconstructive Surgery, Chang Gung Memorial Hospital at Keelung, Chang Gung University and College of Medicine, Keelung, Taiwan; 2 Department of Biomedical Engineering, College of Medicine and College of Engineering, National Taiwan University, Taipei, Taiwan; 3 Department of Surgery, National Taiwan University Hospital and College of Medicine, 7 Chung-Shan S. Rd., Taipei, 100 Taiwan; 4 Department of Medical Research, National Taiwan University Hospital, Taipei, Taiwan; 5 Research Center for Developmental Biology and Regenerative Medicine, National Taiwan University, Taipei, Taiwan

**Keywords:** Stem cell culture, Diabetes, Adipose-derived stem cell, Stem cell transplantation, Tissue regeneration

## Abstract

**Background:**

Application of autologous adipose-derived stem cells (ASC) for diabetic chronic wounds has become an emerging treatment option. However, ASCs from diabetic individuals showed impaired cell function and suboptimal wound healing effects. We proposed that adopting a low-glucose level in the culture medium for diabetic ASCs may restore their pro-healing capabilities.

**Methods:**

ASCs from diabetic humans and mice were retrieved and cultured in high-glucose (HG, 4.5 g/L) or low-glucose (LG, 1.0 g/L) conditions. Cell characteristics and functions were investigated in vitro. Moreover, we applied diabetic murine ASCs cultured in HG or LG condition to a wound healing model in diabetic mice to compare their healing capabilities in vivo.

**Results:**

Human ASCs exhibited decreased cell proliferation and migration with enhanced senescence when cultured in HG condition in vitro. Similar findings were noted in ASCs derived from diabetic mice. The inferior cellular functions could be partially recovered when they were cultured in LG condition. In the animal study, wounds healed faster when treated with HG- or LG-cultured diabetic ASCs relative to the control group. Moreover, higher collagen density, more angiogenesis and cellular retention of applied ASCs were found in wound tissues treated with diabetic ASCs cultured in LG condition.

**Conclusions:**

In line with the literature, our study showed that a diabetic milieu exerts an adverse effect on ASCs. Adopting LG culture condition is a simple and effective approach to enhance the wound healing capabilities of diabetic ASCs, which is valuable for the clinical application of autologous ASCs from diabetic patients.

**Supplementary Information:**

The online version contains supplementary material available at 10.1186/s13287-023-03478-2.

## Background

Diabetes mellitus (DM) has become one of the leading causes of disability, affecting up to 537 million patients worldwide in 2021 [1]. Among the DM-related morbidities, chronic ulcer is one of the most common and costly complications, likely impacting 19–34% of diabetic patients [2]. Diabetic wounds are multifactorial, involving impaired wound epithelialization, angiogenesis and collagen matrix formation [3, 4]. As the prevalence of diabetes keeps on increasing, the medical and socio-economic burden of diabetic wounds are expected to increase accordingly. Currently, several regenerative strategies have been proposed to enhance diabetic chronic wound healing, including mesenchymal stem cells (MSCs), stromal vascular fraction (SVF), platelet rich plasma (PRP), hair follicle stem cells (HFSC), and decellularized extracellular matrix (ECM) [5–8]. Among all these treatment modalities in the field of regenerative plastic surgery, MSCs are regarded to exhibit great potential to promote wound healing [9–11].

Adipose-derived stem cell (ASC) represents an abundant MSC source that can easily be obtained from subcutaneous adipose tissue through minimally invasive surgical intervention, such as liposuction [12–14]. The easy accessibility and low donor site morbidity have made ASCs good candidates for a broad range of cell-based therapeutics. They can be applied alone or in combination with some other biomaterials, such as PRP or hydrogel scaffolds containing hyaluronic acid [7, 11, 15]. Particularly, ASCs secrete several growth factors that stimulate angiogenesis and cell growth, including vascular endothelial growth factor (VEGF), fibroblast growth factor-2 (FGF2) and hepatocyte growth factor (HGF) [4, 16, 17]. ASC-based treatment for chronic diabetic wounds has therefore been gaining its popularity through the mechanism of promoting angiogenesis, immunomodulation and epithelialization [4, 17–19].

In general, employing autologous ASCs for cell therapy exhibit lower regulatory hurdles, and they induce less immune response than allogeneic ASCs [20–23]. Nevertheless, ASCs derived from diabetic donors appeared to exhibit impaired proliferation and angiogenic potential, and less satisfactory outcomes were observed when they were applied for wound healing [24–26]. Our previous study also confirmed lower proliferation and migration capabilities in ASCs derived from diabetic patients [27]. Several previous studies revealed a negative effect of high-glucose environment on ASCs, so it remains a concern that diabetic ASCs may be functionally impaired for wound therapy [28–30]. It is thus important to restore the regenerative capabilities of diabetic ASCs during in vitro expansion process to ensure a better therapeutic effect of autologous ASC application for skin wounds.

In the literature, human MSCs are frequently cultured in high-glucose medium in the literature (~ 4.5 g/L) [31, 32]. However, low-glucose culture medium (~ 1.0 g/L) has also been adopted [33–35]. There is a lack of consensus on the optimal glucose concentration of the culturing condition for ASCs to date [36, 37]. We herein hypothesized that subjecting human ASCs to a medium with low-glucose concentration for cell culture could be beneficial for preserving their regenerative capability. We also explored the characteristics of ASCs from diabetic mice under high- or low-glucose culture environment in vitro, and their wound healing capability was further investigated in vivo using a diabetic murine wound model.

## Methods

### Isolation and culture of human adipose-derived stem cells

The study protocol was approved by the Research Ethics Committee of National Taiwan University Hospital (No. 201303038RINB), and the informed consent was obtained from each donor of adipose tissue. We conducted the study in accordance with the institutional biosafety standards. Human adipose-derived stem cells (hASCs) were extracted from two non-diabetic and two diabetic female donors for the single-cell data processing (serum HbA1c levels were 11.8% and 9.6% for the two diabetic donors). For the subsequent human cell experiments, hASCs previously obtained from another four non-diabetic female donors were used [mean age: 45.3 years (32–57 years); mean body mass index: 24.6 (21.0–26.6)] [38, 39]. These cells were mixed and pooled together as a single population, which have been previously tested to exhibit differentiation capabilities toward osteogenic, chondrogenic and adipogenic lineages [40].

To isolate hASCs, small pieces of subcutaneous fat tissue from each donor were finely minced and washed using phosphate-buffered saline (PBS; Omics Biotechnology, Taipei, Taiwan), followed by type I collagenase (1 mg/mL; Gibco, Carlsbad, California) treatment at 37 °C for 60 min. Fetal bovine serum (FBS; Hyclone, Logan, Utah) was added before the cell suspension was filtered and centrifuged. The pellets were later suspended and plated with Dulbecco’s modified Eagle’s medium/Nutrient Mixture F-12 (DMEM/F-12; Hyclone), supplemented with 10% FBS, 1% penicillin–streptomycin (Biological Industries, Kibbutz Beit Haemek, Israel), and 1 ng/mL FGF2 (R&D systems, Minneapolis, Minnesota). The cells were then cultured in a 5% CO_2_ humidified atmosphere at 37 °C, and the medium was changed every 2–3 days. Upon reaching 90% confluence, the cells were detached using 0.05% trypsin–EDTA (Biological Industries) and replated until the P3 or P4 for various experiments.

### Single-cell data processing of hASCs

The hASCs used in the single-cell RNA sequencing (RNA-Seq) analysis from two non-diabetic and two diabetic donors were labeled as control and DM groups, respectively. Each hASC was isolated using Single-Cell Capture and cDNA Synthesis with the BD Rhapsody Express Single-Cell Analysis System (BD Biosciences, Franklin Lakes, New Jersey) following the manufacturer’s protocol, and we prepared the single-cell libraries using BD Rhapsody Whole Transcriptome Analysis (WTA) and BD Single-Cell Multiplexing Kits (BD Biosciences). Final libraries were multiplexed for paired-end (150 bp) sequencing on a HiSeq X sequencer (Illumina, San Diego, California). Fastq files were processed via the standard Rhapsody analysis pipeline (BD Biosciences) on Seven Bridges (https://www.sevenbridges.com) per the manufacturer’s recommendations. Recursive substation error correction (RSEC) was utilized for correcting polymerase chain reaction and sequencing errors, which was developed by the manufacturer. The R package Seurat was utilized for all downstream analysis.

### Characteristics of hASCs cultured in high-/low-glucose conditions

To simulate a non-diabetic or diabetic environment in vitro, non-diabetic hASCs were maintained in DMEM-low glucose for 49 days (DMEM-LG, 1 g/L; labeled as 49L group), DMEM-high glucose for 49 days (DMEM-HG, 4.5 g/L; labeled as 49H group), or DMEM-HG for 35 days, followed by 14 days in DMEM-LG (labeled as 35H14L group). The medium consisted of 10% FBS and 1% penicillin–streptomycin and were changed every 2–3 days. The cells were lifted and replated at a density of 9000 cells/cm^2^ every 7 days as a new passage, and the cumulative population doubling was calculated at each passage.

After 49 days, the characteristics of hASCs from each culture condition were tested. Proliferative activity of hASCs was estimated with a cell proliferation enzyme-linked immunosorbent assay kit (ELISA; Roche, Indianapolis, Indiana) involving 5-bromo-2′-deoxyuridine (BrdU). The cells were incubated with BrdU for 24 h, and the anti-BrdU-peroxidase was added and bound to the BrdU. The formed immune complexes were detected and quantified by an ELISA reader to estimate the DNA synthesis amount, indicating the proliferative activity.

To investigate the survival of hASCs under stress in each culture condition, they were further incubated in serum-free medium for 7 days. A Cytotoxicity Detection Kit (Roche) was adopted to determine the levels of lactate dehydrogenase (LDH) released by dead cells into the medium. To evaluate the cellular aging, hASCs were fixed in 4% paraformaldehyde (MilliporeSigma, St. Louis, Missouri) at room temperature for 8 min and stained with the senescence-associated β-galactosidase (SA-β-Gal) chromogenic substrate solution (MilliporeSigma). The proportion of SA-β-Gal-positive cells in randomly selected high-power fields was calculated.

Moreover, cell migratory ability in each culture condition was assessed by an in vitro scratch assay. The hASCs were seeded in 24-well culture plates (Corning, Corning, New York). Upon cell confluence, the well surface was gently scratched with a 200 μL sterile pipette tip to create a rectangular cell-free zone. After the culture medium and scraped cells were removed, the remaining cells were washed twice with PBS and cultured for 24 h. The cell images were obtained at 0, 12, and 24 h under an inverted microscope (Nikon, Tokyo, Japan) and analyzed with ImageJ.

### Isolation and characterization of murine diabetic ASCs

Male BKS.Cg-*Dock*^*7m*^+/+*Lepr*^*db*^/JNarl (db/db) mice (30–40 g body weight and 6–8 weeks old; National Laboratory Animal Center, Taipei, Taiwan) were used in the animal experiments, which were conducted in compliance with the Institutional Animal Care and Use Committee (IACUC) guidelines of National Taiwan University College of Medicine. Only mice with blood glucose levels greater than 300 mg/dL were considered diabetic, and ASCs were obtained from the subcutaneous fat tissue in some of these mice. The cell isolation process was similar to that of hASCs. ASCs derived from db/db mice, referred as dbASCs, were cultured in minimum essential medium alpha modification (α-MEM; Hyclone), supplemented with 10% FBS, 1% penicillin–streptomycin, and 1 ng/mL mouse FGF2 (BioLegend, San Diego, California). At P2, the culture medium was either shifted to high-glucose (α-MEM-HG, 4.5 g/L) or low-glucose (α-MEM-LG, 1 g/L) condition for 14 days, in which the dbASCs were labeled dbHG or dbLG, respectively. Then, the murine ASCs were trypsinized to produce single-cell suspensions for analysis of cell surface antigen expression using a flow cytometer (FACSVerse, BD Biosciences) and its software (FACSuite, BD Biosciences), which counted 10^5^ cells per sample. Antibodies used for detecting ASC surface markers included anti-CD31 (BD Biosciences), anti-CD34 (BioLegend), and anti-CD90 (BioLegend). The positive cells were defined as those with fluorescence > 95% of the signal of the isotype controls.

The adipogenic and osteogenic potential of the isolated dbASCs were also examined. For adipogenesis, confluent cells were cultured in α-MEM, supplemented with 10% FBS, 10 µM insulin, 200 µM indomethacin (MilliporeSigma), 500 µM 3-isobutyl-1-methylxanthine (IBMX; MilliporeSigma), and 1 µM dexamethasone (Dex; MilliporeSigma). After 2 weeks of induction, the adipogenic capability of the dbASCs was estimated by Oil Red O staining. For osteogenesis, cells were cultured in α-MEM, supplemented with 10% FBS, 10 nM Dex, 10 mM β-glycerophosphate disodium salt hydrate (β-GP; MilliporeSigma), 50 µM l-ascorbic acid (Vit C; MilliporeSigma), and 10 nM 1α,25-dihydroxycholecalciferol (Vit D3; MilliporeSigma). After 3 weeks of induction, the osteogenic capability of the dbASCs was investigated by Alizarin Red S staining.

### Proliferation and senescence of dbASCs in high/low-glucose conditions

Every 7 days, dbASCs subjected to HG or LG culture were detached by 0.05% trypsin–EDTA and replated at a density of 2500 cells/cm^2^, and the cumulative population doubling was calculated at each passage. At P4, P6, P8, and P10, the cells were stained with SA-β-Gal, and the proportion of positively stained cells was calculated. Moreover, reactive oxygen species (ROS) level was detected utilizing the ROS-ID® Total ROS/Superoxide Detection Kit (Enzo Life Sciences, Farmingdale, New York). The dbASCs were seeded at 4000 cells/well and loaded with 100 µL/well of oxidative stress detection reagent from the kit at 37 °C for 60 min. ROS level was determined by the optical density using a fluorescence microplate reader (Tecan, Männedorf, Switzerland) at the excitation and emission wavelengths of 488/520 nm, respectively.

### Quantitative polymerase chain reaction (qPCR)

Total RNA was extracted from dbASCs in HG or LG culture conditions with RNeasy Mini Kit (Qiagen, Germantown, Maryland) and reverse-transcribed to complementary DNA (cDNA) with the High-Capacity cDNA Reverse Transcription Kit (ThermoFisher, Waltham, Massachusetts). Real-time qPCR was performed with iQ SYBR Green Supermix (Bio-Rad, Hercules, California) using the CFX Connect Real-Time PCR detection system and CFX Manager (Bio-Rad). We normalized the expression level to glyceraldehyde 3-phosphate dehydrogenase (GAPDH) for each cDNA sample and calculated the relative quantity of gene expression using dbHG samples as references. The targeted genes and their primer sequences were described in the Additional file 1: Table S1.

### Conditioned medium of dbASCs for endothelial cell and fibroblast culture

After replaced with serum-free medium for 48 h, conditioned medium (CM) of HG- or LG-cultured dbASCs was collected and referred as CM-dbHG and CM-dbLG, respectively. The concentration of secreted VEGF and HGF in CM was estimated using the relevant ELISA kits (R&D Systems). Data were normalized per 10^5^ cells at the time of harvest.

CM-dbHG and CM-dbLG were further used to culture human umbilical vein endothelial cells (HUVECs). Proliferative activity of HUVECs cultured in the respective CM was evaluated with the BrdU assay as described previously. For the in vitro tube formation assay, we seeded HUVECs on *μ*-slides (Ibidi, Gräfelfing, Germany) coated with Matrigel (Corning) at a density of 7500 cells/well. Culture media with a mixture of endothelial growth medium 2 (EGM2; PromoCell, Heidelberg, Germany) and CM-dbHG or CM-dbLG at a volume ratio of 1:1 were then applied. Endothelial basal medium 2 (EBM2; PromoCell) and EGM2 served as negative and positive controls, respectively. Formation of tube-like structures under a phase-contrast microscope was analyzed using ImageJ after 6 h of culture.

Moreover, human dermal fibroblasts (ScienCell, Carlsbad, California) were seeded at a density of 10^5^ cells/well and cultured in CM-dbHG or CM-dbLG for 24 h. The migratory capability of fibroblasts in CM-dbHG and CM-dbLG was also assessed by the in vitro scratch assay as described previously for hASCs.

### In vivo murine wound healing model

For the in vivo wound healing model, db/db mice were anesthetized by intraperitoneal injection of a mixture of zolazepam (Zoletil vet, Virbac, Taipei, Taiwan) and xylazine (Rompun, Bayer, Germany; 4:1, v/v). Two identical, circular, full-thickness cutaneous wounds in a diameter of 13 mm were surgically created on each side of the dorsum. Each wound margin was splinted with an O-ring by sutures to prevent the wounds from contraction. In each mouse, the wound on the left side was applied with 100 μL PBS as controls, and the wound on the right side was treated with 5 × 10^5^ ASCs (dbHG or dbLG) suspended in 100 μL PBS. A transparent, semiocclusive adhesive dressing (Tegaderm; 3 M, St. Paul, Minnesota) was applied over the wounds for protection. The wounds were photographed regularly postoperatively, and the wound size was measured through planimetric methods using ImageJ. The db/db mice were euthanized by 50% CO_2_ and harvested entire wound tissues, including some of the surrounding skin area, on the postoperative days 5 and 21 for further analysis (*n* = 3 for each time point in each group).

### Histology, immunohistochemistry, and dbASC retention

Wound samples were fixed in 4% paraformaldehyde, embedded with paraffin, sectioned into slides, and stained with hematoxylin and eosin (H&E; MilliporeSigma) or Masson’s trichrome (MilliporeSigma) per the manufacturer’s protocols. We measured the collagen density in three randomly selected regions of interest on Masson’s trichrome-stained sections by setting color threshold and quantifying collagen proportion with ImageJ [41]. For immunohistochemical staining, wound sections were deparaffinized with Trilogy (Cell Marque, Rocklin, California) and labeled using antibodies against CD31, CD68, and α-SMA (all from Abcam, Cambridge, United Kingdom). Subsequent staining was done according to the manufacturer’s protocols of the TnAlink Polymer Detection System Kit (BioTnA, Kaohsiung, Taiwan), which included secondary antibodies with polymer-conjugated horseradish peroxidase that reacted with mouse and rabbit primary antibodies. Positively stained cells were counted under randomly selected high-power fields (hpf).

To investigate the retention of transplanted dbASCs within the recipient wound bed, Qtracker Cell Labeling Kit (ThermoFisher) was used to label HG- and LG-cultured dbASC according to the protocol provided by the manufacturer. We applied 5 × 10^5^ Qtracker-labeled cells to each wound of the db/db mice as previously described (*n* = 3). The mice were sacrificed on day 5, and positively stained cells within randomly selected hpf were counted under a fluorescence microscope.

### Statistical analysis

The data were expressed as the mean ± SD and tested to meet the assumptions of normality and homogeneity of variance when applicable. Unpaired *t* tests were used for comparison between two different groups, and ANOVA followed by Tukey’s post-hoc tests was utilized for comparison among three or more groups. All statistical analyses were performed using GraphPad Prism version 6.0 software. The R package Seurat was used for single-cell RNA sequence data analysis. Statistically significant values were defined as *p* < 0.05.

## Results

### Single-cell RNA-Seq analysis of hASCs

**Fig. 1:**
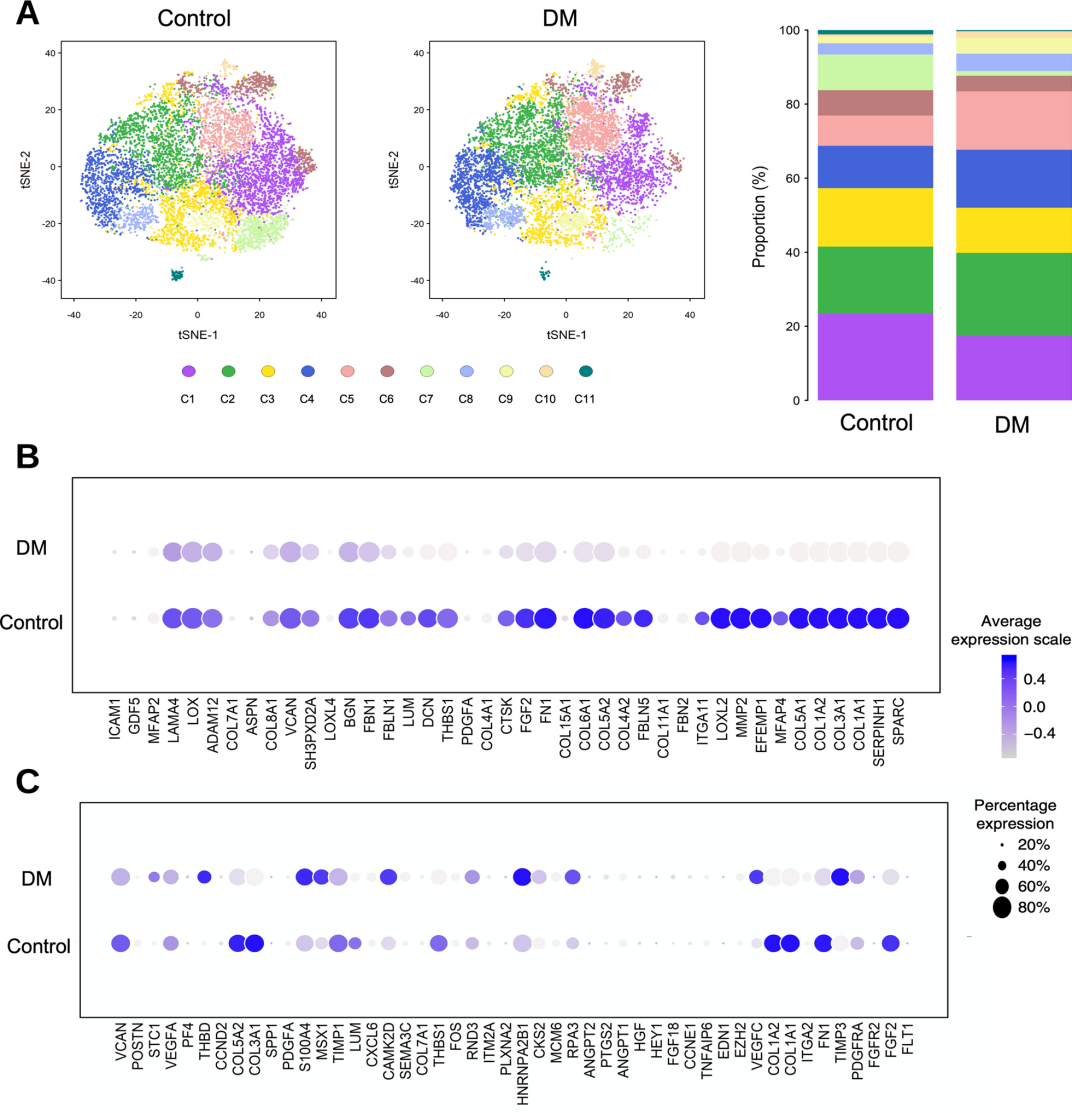
Single-cell RNA sequencing analysis of hASCs from non-diabetic (control) and diabetic (DM) patients. **A** Cell population distribution by t-SNE projections showed a similar pattern in control and DM individuals (left and middle, *n* = 8812 and 8689, respectively). Bar charts of 11 clusters identified from control and diabetic ASCs showed slight differences in the proportion of clusters (right). **B** Dot plot showed decreased expression of the several ECM organizing-related genes in hASCs from DM patients. **C** Dot plot showed variation in the relative expression of angiogenesis-related genes in hASCs from both groups. The sizes of the dots in (**B**) and (**C**) corresponded to the percentage of cells expressing the genes in each cluster, and the color scale represented the average expression level. *hASC* human adipose-derived stem cell, *ECM* extracellular matrix, *t-SNE* t-distributed stochastic neighbor embedding

The single-cell RNA-Seq analysis reveals differential expression level of genes and provides information about functional properties of various cell subsets within a heterogenic cell cluster. We adopted the technique to identify global changes in gene expression of hASCs from diabetic when compared to non-diabetic individuals. Hierarchical clustering and *t*-distributed stochastic neighbor embedding (*t*-SNE) projection on all 17,501 hASCs (8812 control cells and 8689 DM cells) resulted in 11 subgroups of cells with similar distribution in the respective groups depending on the expression patterns (Fig. 1A, left), and the proportion of each subpopulation revealed slight differences between the two groups (Fig. 1A, right). The raw data were submitted online to the Gene Expression Omnibus (GEO) and are open to public.

To investigate the difference in the biological pathways between DM and control hASCs, we grouped genes from each Gene Ontology (GO) term as a unit to perform Gene Set Enrichment Analysis (GSEA). Our analysis revealed an enrichment in the ECM organization gene set in the control group relative to the DM group. In 41 gene signatures related to ECM organization, most of the genes were significantly upregulated in the control group compared to the DM group, such as *FGF2*, *FN1*, *COL1A1*, *COL1A2*, *COL3A1*, and *MMP2* (Fig. 1B). Meanwhile, variable changes in the relative gene expression among angiogenesis-related genes were noted (Fig. 1C).

### Influence of glucose concentration on hASCs

**Fig. 2:**
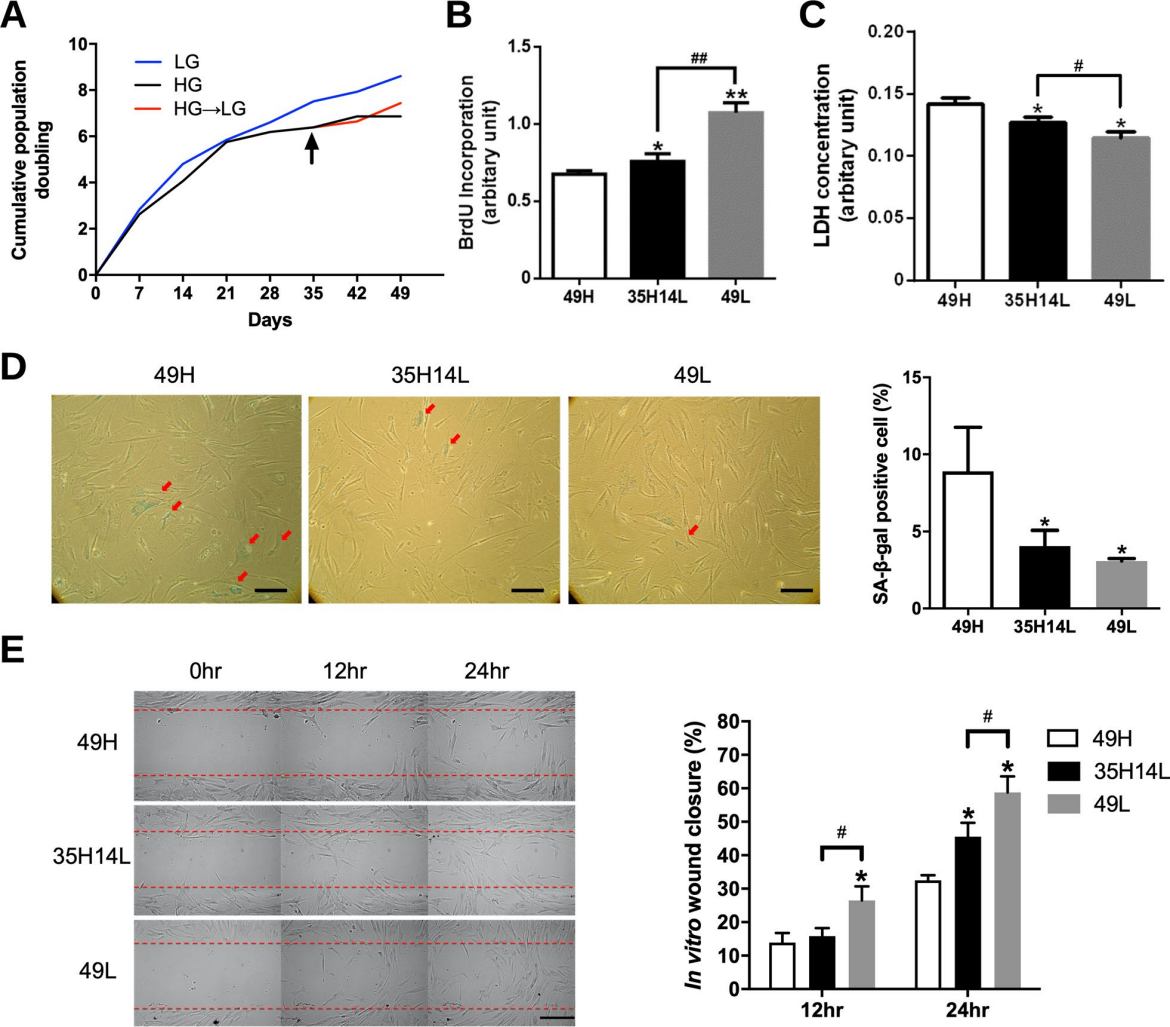
Characteristics of control hASCs cultured in high- or low-glucose conditions. **A** Cumulative population doubling of hASCs revealed a lower proliferative activity when cultured in HG condition, and the cell growth could be enhanced after shifting to LG condition at day 35 (arrow). **B** Proliferative activity estimated by BrdU incorporation showed faster growth of hASCs in the 35H14L and 49L groups. **C** Cytotoxicity of hASCs in the serum-free condition determined by the LDH efflux assay. **D** SA-β-gal staining of hASCs and their quantification in five randomly selected power fields. Scale bar = 100 μm. **E** Cell migration of hASCs evaluated by in vitro scratch assay at different time points. Scale bar = 100 μm. *p < 0.05, **p < 0.01 relative to the 49H group; ^#^*p* < 0.05, ^##^*p* < 0.01 between the indicated groups. *BrdU* 5-bromo-2′-deoxyuridine, *hASC* human adipose-derived stem cell, *HG* high glucose, *LG* low glucose, *hpf* high-power field, *SA-β-gal* senescence-associated β-galactosidase, *49H* 49 days of high-glucose condition, *49L* 49 days of low-glucose condition, *35H14L* 35 days of high-glucose condition followed by 14 days of low-glucose condition

In the population doubling study, hASCs under long-term LG culture condition (49L group) exhibited higher proliferative activity than those subjected to long-term HG culture (49H group). Shifting hASCs in HG condition to LG condition at day 35 (35H14L group) improved their proliferative activity compared to those constantly maintained in HG culture condition (Fig. 2A). The observation was confirmed by the BrdU incorporation assay at day 49, which revealed significantly decreased proliferation rate in the 49H group when compared to the 49L group (*p* < 0.01) and the 35H14L group (*p* < 0.05; Fig. 2B). LDH concentration in the medium was examined for evaluation of cytotoxicity under serum-free conditions, and hASCs in the 49H group exhibited a significantly higher level of LDH release relative to the other two groups (*p* < 0.05; Fig. 2C).

SA-β-gal staining was applied to determine the senescent hASCs in each group. When examined under a microscope, some flattened and round-shaped cells could be identified with blue stains in each group (Fig. 2D, left, indicated by red arrows). The 49H group exhibited significantly more positively stained cells when compared to the other two groups (49H: 8.9 ± 2.9% vs. 35H14L: 4.1 ± 1.0%, *p* < 0.05; vs. 49L: 3.1 ± 0.2%, *p* < 0.05; Fig. 2D, right).

The in vitro scratch assay is a rather simple and economical method for study of collective cell migration, which simplifies and simulates the critical movement in the process of wound healing. In the assay, the coverage of migrated hASCs over the scratched areas was observed and measured via captured images (Fig. 2E, left). The 49H group exhibited a significantly smaller wound closure ratio relative to the other two groups at 24 h (49H: 32.5 ± 1.5% vs. 35H14L: 45.6 ± 4.1%, *p* < 0.05; vs. 49L: 58.7 ± 4.8%, *p* < 0.05), while the 35H14L group also showed a significantly smaller closure ratio compared to the 49L group (*p* < 0.05; Fig. 2E, right).

### Phenotypic characterization of dbASCs in HG and LG condition

**Fig. 3:**
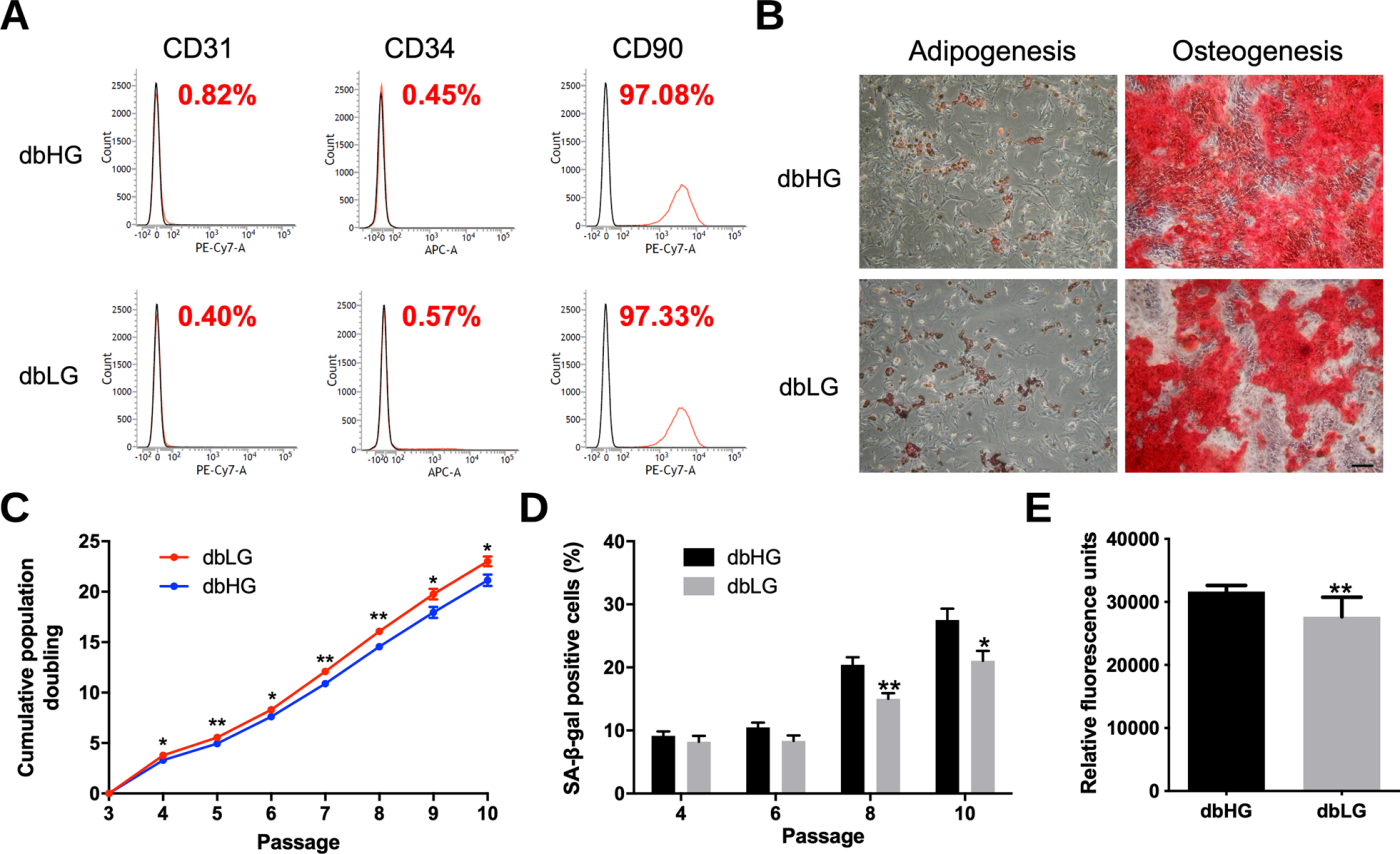
Phenotypic characterization of murine ASCs from db/db mice in high-glucose (dbHG) or low-glucose (dbLG) conditions. **A** The expression level of ASC surface markers is shown as the proportion of positively stained cells relative to the isotype control. Both groups of ASCs were negative for the hematopoietic markers CD31 and CD34 but positive for the mesenchymal stem cell-related markers CD90. **B** Murine ASCs were cultured in adipogenic or osteogenic induction medium, and cells were stained with Oil Red O for the detection of adipogenesis and Alizarin Red for the detection of osteogenesis. Scale bar = 200 μm. **C** Cumulative population doubling curve showing faster proliferation of the dbLG group. **D** Quantification of SA-β-gal-positive cells in five randomly selected power fields revealed more senescent cells in the dbHG group. **E** After cultured in LG and HG conditions, murine ASCs were examined for ROS production. Significantly more ROS production was noted in the dbHG condition. *p < 0.05, **p < 0.01 relative to dbHG. *ASC* adipose-derived stem cell, *SA-β-gal* senescence-associated β-galactosidase, *ROS* reactive oxygen species

At 14 days of culture, the flow cytometer revealed that the surface epitopes of the HG-treated dbASCs (dbHG) were similar to those of the LG-treated dbASCs (dbLG). These two groups of ASCs were both negative for the endothelial marker CD31, the hematopoietic marker CD34, and positive for the MSC-associated marker CD90 (Fig. 3A). Adipogenic and osteogenic potential after applying appropriate induction media were maintained in both groups, as demonstrated by Oil Red O and Alizarin Red S staining, respectively (Fig. 3B). In the cumulative population doubling study, significantly higher proliferation rate was found in the dbLG group compared to the dbHG group from P4 to P10 (Fig. 3C).

Senescence of dbASCs in HG and LG culture condition at different passages was analyzed using SA-β-gal staining, revealing significantly more positively stained cells in the dbHG group at P8 and P10 (P8: 20.4 ± 4.6% vs. 15.0 ± 3.5%, *p* < 0.01; P10: 27.5 ± 6.9% vs. 21.0 ± 6.2%, *p* < 0.05; Fig. 3D). Intracellular ROS level was detected to assess the oxidative stress of dbASCs in different culture environment. In the ROS detection assay, the dbHG group exhibited a significantly higher intracellular ROS level relative to the dbLG group (*p* < 0.01; Fig. 3E).

### Angiogenic growth factor expression of dbASCs in HG and LG condition

**Fig. 4:**
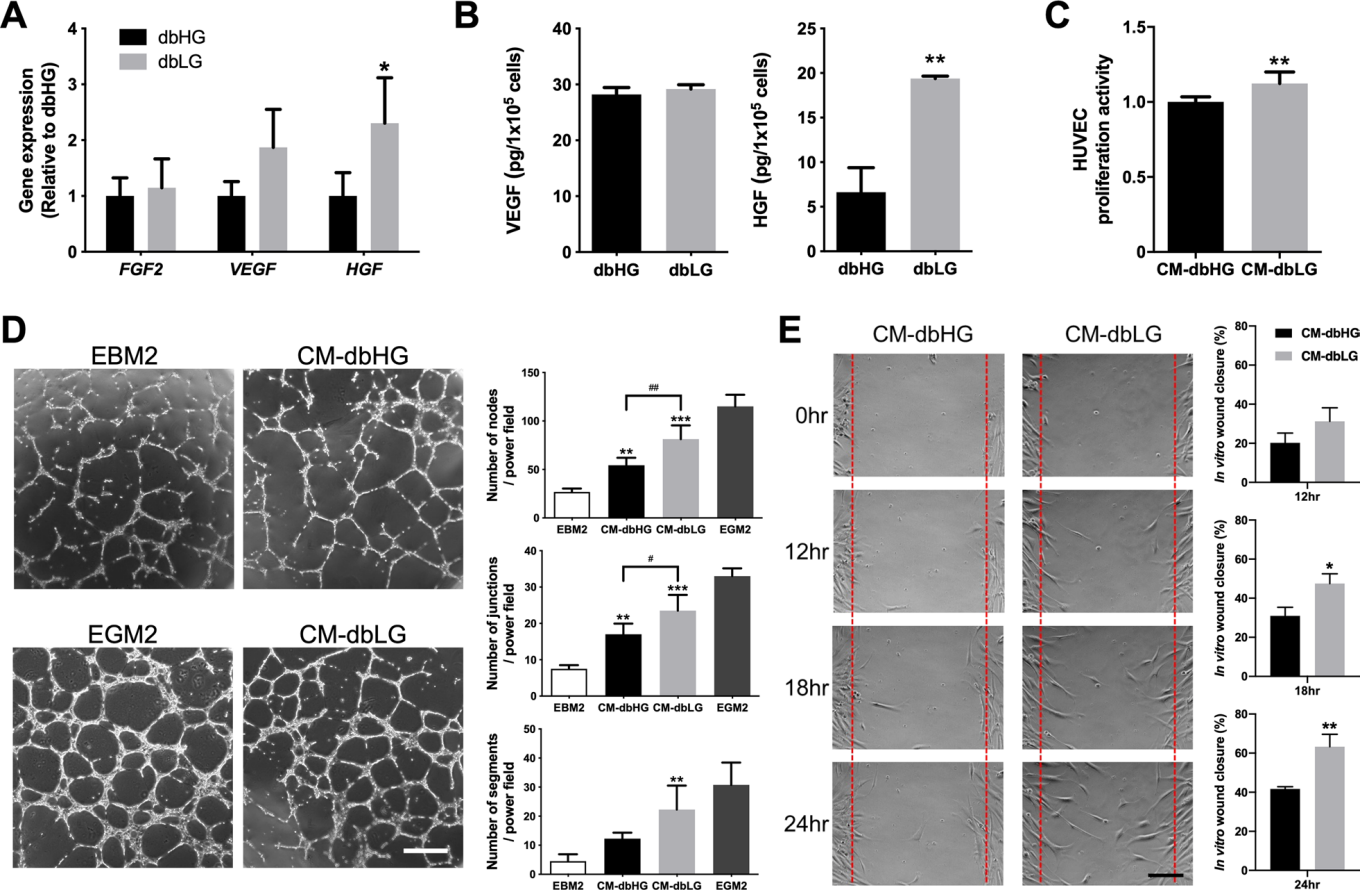
Expression of angiogenic growth factors in dbASCs under high-glucose (dbHG) and low-glucose (dbLG) conditions. **A** Evaluation of angiogenesis‑related gene expression by quantitative RT-PCR, including *FGF2*, *VEGF*, and *HGF*. Values are normalized by GAPDH expression level and plotted as relative quantity to the dbHG group. *p < 0.05 relative to dbHG. **B** ELISA showed significantly higher concentration of HGF in CM-dbLG. *p < 0.05 relative to CM-dbHG. **C** BrdU assay demonstrated enhanced proliferative activity of HUVECs incubated in CM-dbLG. **p < 0.01 relative to CM-dbHG. **D** In vitro tube formation of HUVECs. HUVECs cultured in endothelial growth medium 2 (EGM2) served as positive controls, and those in endothelial basal medium 2 (EBM2) were negative controls. Scale bar = 500 μm. Branching nodes, junctions and segments per power field were compared among different groups except the positive control. *p < 0.05, **p < 0.01 relative to control; ^#^*p* < 0.05, ^##^*p* < 0.01 between the indicated groups. **E** In vitro scratch assay of human dermal fibroblasts. Red dotted lines represent the initial borders of the cell-free zone. Scale bar = 200 μm. Quantification of the wound closure percentage of fibroblasts was shown. *p < 0.05, **p < 0.01 relative to CM-dbHG. *dbASC* murine ASCs from db/db mice, *CM-dbHG* conditioned medium from dbASCs cultured in HG condition, *CM-dbLG* conditioned medium from dbASCs cultured in LG condition, *HUVEC* human umbilical vein endothelial cell

Real-time qPCR was employed to evaluate the expression level of *FGF2*, *VEFG*, and *HGF* in dbASCs under HG and LG conditions, and *HGF* was significantly upregulated in the dbLG group relative to the dbHG group (2.3 ± 0.8-fold upregulation, *p* < 0.05; Fig. 4A). Further ELISA analysis of VEGF and HGF in the CM derived from dbHG and dbLG also confirmed a significantly higher concentration of HGF in CM-dbLG relative to CM-dbHG (19.4 ± 0.3 vs. 6.6 ± 2.7 pg/10^5^ cells, *p* < 0.01; Fig. 4B).

CM harvested from dbHG and dbLG was used for HUVEC culture, and BrdU assay showed significantly higher proliferation rate when HUVECs were cultured in CM-dbLG relative to CM-dbHG (*p* < 0.01; Fig. 4C). Moreover, in vitro tube formation of HUVECs under the influence of different CM was evaluated via quantification of nodes, junctions, and segments per power field. The CM-dbLG group exhibited significantly more nodes (81.3 ± 14.2 vs. 54.3 ± 7.8 nodes/hpf, *p* < 0.01) and junctions (23.5 ± 4.4 vs. 17.0 ± 2.9 junctions/hpf, *p* < 0.05) relative to the CM-dbHG group, and only the CM-dbLG group exhibited significantly more segments relative to the control group (22.3 ± 8.3 vs. 4.5 ± 2.4 segments/hpf, *p* < 0.01; Fig. 4D). As for the in vitro scratch assay, human dermal fibroblasts cultured in the CM-dbLG showed a significantly higher ratio of wound closure relative to the CM-dbHG group (at 18 h: 47.5 ± 5.0% vs. 31.0 ± 4.4%, *p* < 0.05; at 24 h: 63.3 ± 6.3% vs. 41.7 ± 1.2%, *p* < 0.01; Fig. 4E).

### Transplantation of dbASCs for in vivo wound healing

**Fig. 5:**
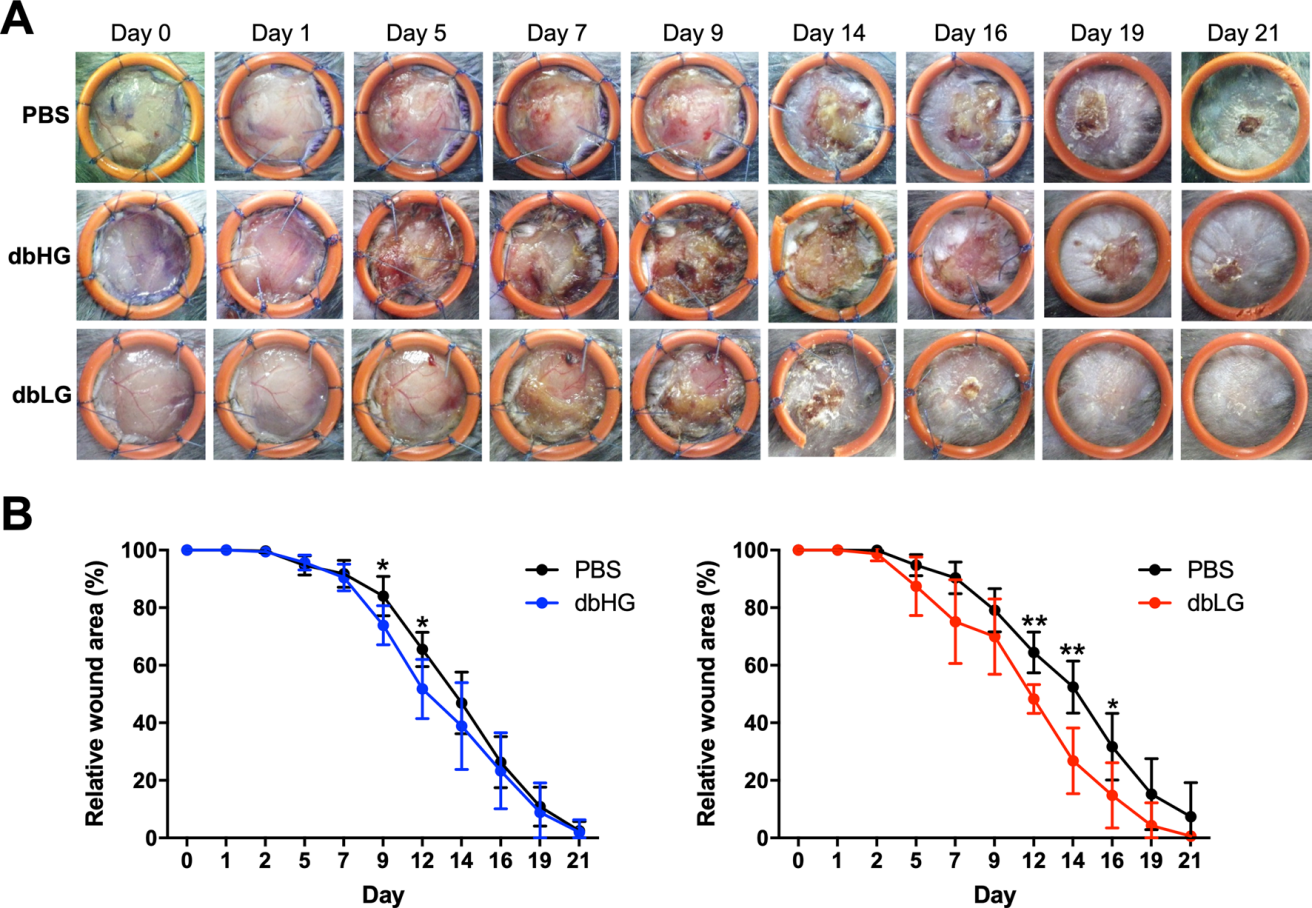
Application of murine ASCs from db/db mice in high-glucose (dbHG) or low-glucose (dbLG) conditions in a diabetic wound healing model. **A** Gross pictures of wounds applied with PBS, dbHG, or dbLG. **B** Relative wound area curves of the dbHG and dbLG groups compared with the contralateral wounds that received PBS treatment. *p < 0.05, **p < 0.01 relative to the PBS group. *dbASC* murine ASCs from db/db mice, *dbHG* dbASCs cultured in HG condition, *dbLG* dbASCs cultured in LG condition

The influence of dbHG and dbLG on diabetic wound healing was evaluated in vivo using a dorsal wound model in db/db mice (Fig. 5A). To obtain more accurate results, we grouped dbHG or dbLG-treated wounds separately and compared them to their contralateral control wounds. Diabetic wounds treated with dbHG exhibited significantly smaller wound area ratio relative to those treated with PBS on day 9 (73.9 ± 6.8% vs. 84.1 ± 6.8%, *p* < 0.05) and day 12 (51.8 ± 10.3% vs. 65.5 ± 6.0%, *p* < 0.05; Fig. 5B, left). Wounds treated with dbLG were also significantly smaller relative to the control group on day 12 (48.3 ± 5.0% vs. 64.4 ± 7.1%, *p* < 0.01), day 14 (26.8 ± 11.4% vs. 52.4 ± 9.1%, *p* < 0.01) and day 16 (14.8 ± 11.3% vs. 31.7 ± 11.6%, *p* < 0.05; Fig. 5B, right).

### Histology, immunohistochemistry, and dbASC retention in the wound tissue

**Fig. 6:**
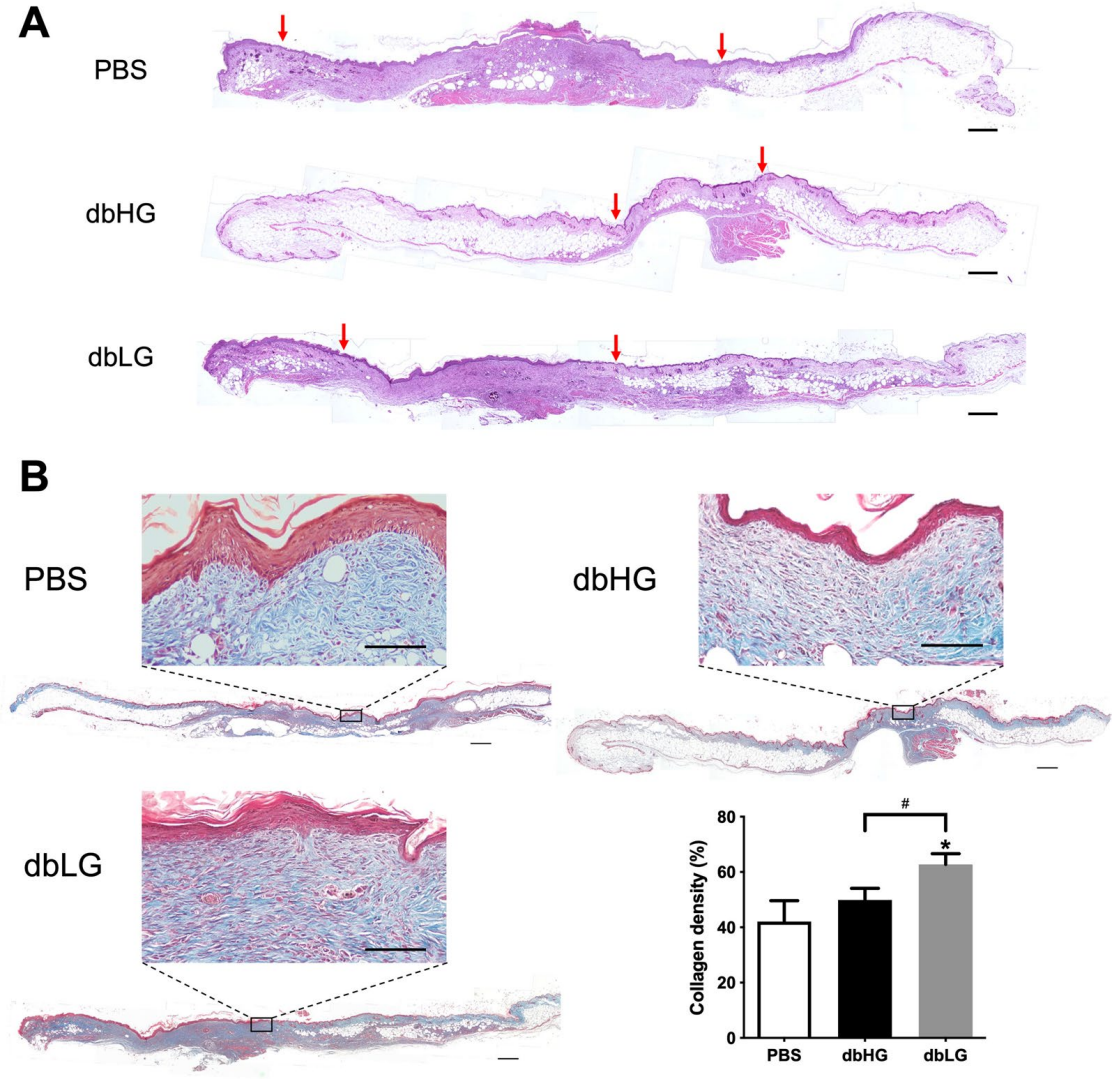
Histology of the healed wound tissue. **A** Representative images of H&E-stained wound sections at day 21. Red arrows indicated the wound margin. Scale bar = 500 µm. **B** Representative images of Masson’s trichrome-stained wound sections at day 21. The differences in the dermal matrix organization were examined under higher magnification, and wounds treated with dbLG exhibited significantly higher collagen density relative to those received PBS or dbHG. Scale bar = 500 µm; higher magnification scale bar = 100 µm. *p < 0.05 relative to PBS, ^#^*p* < 0.05 between the indicated groups. *dbASC* murine ASCs from db/db mice, *dbHG* dbASCs cultured in HG condition, *dbLG* dbASCs cultured in LG condition, *H&E* hematoxylin and eosin

H&E staining was used for detailed analysis of the architecture of wound tissue (Fig. 6A; wound margins indicated by red arrows). Masson’s trichrome staining allowed visualization and quantification of collagen deposition of the wound area in samples from all groups on day 21. Collagen density was measured by means of calculating proportion of blue-stained area in regions of interest with ImageJ, and the dbLG group exhibited a significantly higher collagen density (62.7 ± 3.9%) when compared to the other two groups (dbHG: 49.9 ± 4.2%, PBS: 42.1 ± 7.6%, *p* < 0.05 respectively; Fig. 6B), while the dbHG group showed no significant difference relative to the control group.

**Fig. 7:**
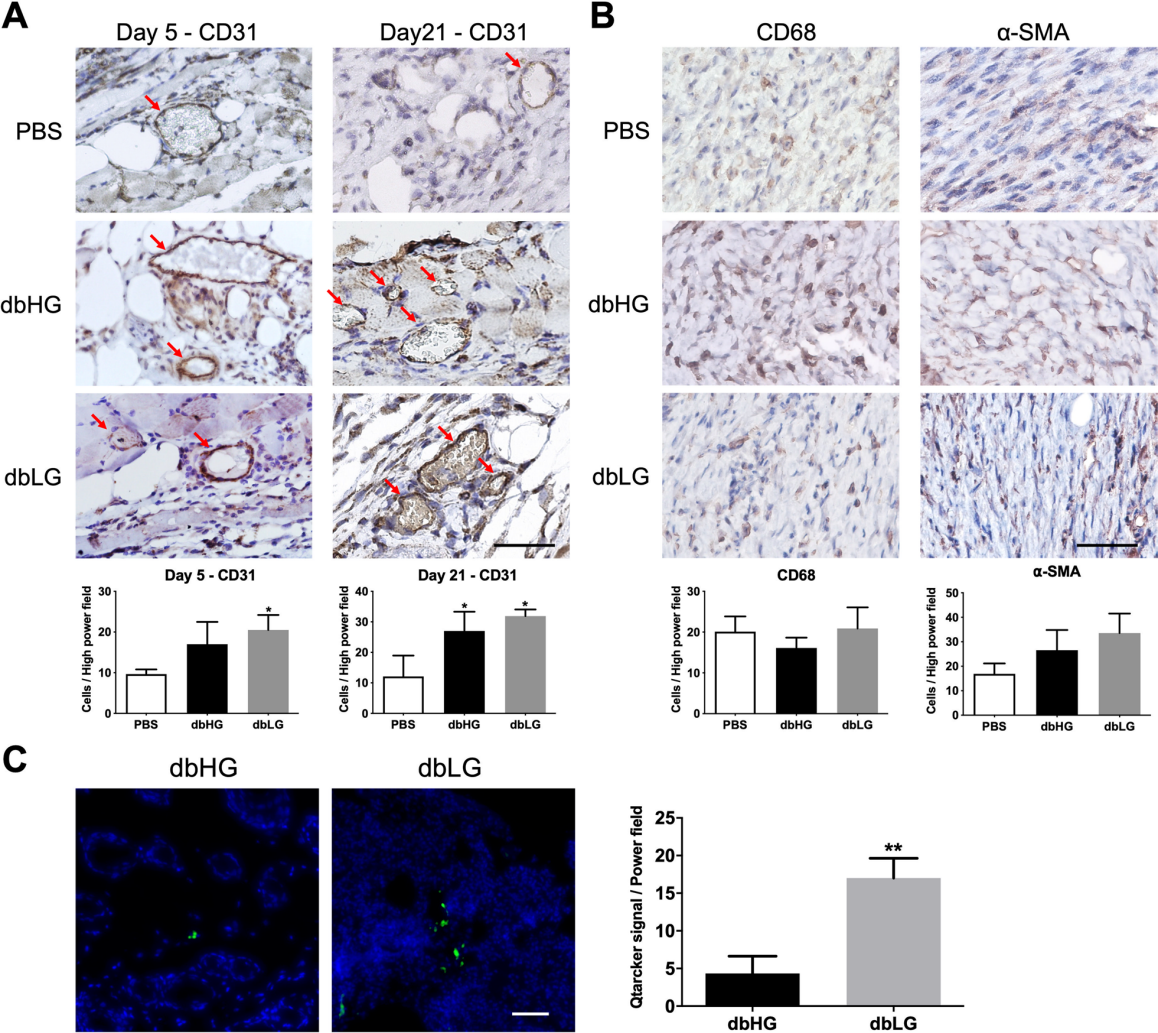
Immunohistochemistry and dbASC retention in the wound tissue. **A** Representative images of the CD31 immunohistochemical sections of wound explants at day 5 and day 21. Red arrows indicate CD31-expressing cells around small vessels. Quantitative analysis of the CD31-expressing cells in high-power field images was shown. Scale bar = 50 µm. *p < 0.05 relative to PBS. **B** Representative images of wound sections stained with anti-CD68 and anti-α-SMA at day 21. Scale bar = 50 µm. Quantitative analysis of the immunohistochemical signals of CD68 and α-SMA showed no differences among different groups. **C** Residual dbASCs in the wound tissues at day 5 were visualized by Qtracker labeling. Scale bar = 100 µm. Significantly more retaining dbASCs were found in the wound tissue treated with dbLG. **p < 0.01 relative to the dbHG group. *dbASC* murine ASCs from db/db mice, *dbHG* dbASCs cultured in HG condition, *dbLG* dbASCs cultured in LG condition

The wound healing effect of dbASCs may be mediated through angiogenesis, anti-inflammation, and antifibrosis mechanisms. Therefore, immunohistochemical staining of CD31, CD68, and α-SMA of the wound tissues was performed. Quantitative analysis of the staining signal revealed significantly more CD31-positive cells in the dbLG group relative to the PBS group on day 5 (20.5 ± 3.7 vs. 9.7 ± 1.2 cells/hpf, *p* < 0.05) and day 21 (31.9 ± 2.2 vs. 12.1 ± 6.8 cells/hpf, *p* < 0.05), while the dbHG group exhibited significantly more CD31-positive cells relative to the PBS group only on day 21 (27.0 ± 6.3 vs. 12.1 ± 6.8 cells/hpf, *p* < 0.05; Fig. 7A). On day 21, the number of cells expressing CD68 and α-SMA in the wound tissue showed no significant differences among three groups (Fig. 7B). Moreover, Qtracker labeling was employed to investigate the retention of transplanted dbASCs in the wound tissues (Fig. 7C, left). On day 5, significantly more dbASCs were found within randomly selected power fields in the dbLG group relative to the dbHG group (17.0 ± 2.7 cells/hpf vs. 4.3 ± 2.3 cells/hpf, *p* < 0.01; Fig. 7C, right).

## Discussion

In the literature, senescence and cell death are found more frequently among ASCs cultured in medium with a HG concentration, and impaired cell proliferation was also noted [36, 37]. To date, several methods have been proposed to regain the cell functions and regenerative effects of diabetic ASCs. For instance, hypoxic stimuli were found to enhance the proliferation and growth factor secretion of diabetic ASCs [24, 29]. Other attempts included pretreatment with a mitochondrially targeted antioxidant [42] or lipopolysaccharide [43], application of ASCs in a SDF-1α gene-activated scaffold [44, 45] or hyaluronic acid scaffold [15, 46], and combining ASC treatment with PRP [6, 7, 11]. In this study, we discovered that the impaired proliferative and migratory abilities of diabetic ASCs could be partially restored along with decreased senescence by subjecting them to LG culture condition in vitro. The superior performance of diabetic ASCs expanded in LG culture medium was further supported by our in vivo findings in a diabetic murine wound model. Despite that both LG and HG-cultured diabetic ASCs could enhance wound healing, the wound tissues in the dbLG group exhibited significantly more angiogenesis and retaining ASCs at day 5 and a significantly higher collagen index at day 21. Therefore, adopting LG culture condition is a simple and effective approach to enhance the wound healing capabilities of diabetic ASCs, which is valuable for clinical application of autologous ASCs in DM patients for regenerative or therapeutic purposes.

We explored the differences in gene expressing patterns between diabetic and non-diabetic hASCs with single-cell RNA-Seq technique. ASCs [47, 48] or SVF [49, 50] derived from adipose tissue of diabetic or non-diabetic individuals have been found to exhibit a distinct pattern of cell subpopulation distribution on the t-SNE 2D cell map. For instance, an enriched cluster of subcutaneous adipocyte progenitors was identified in DM patients, and the cluster proportion was found to be positively correlated with the donors’ fasting glucose level. The differential gene expression analysis also confirmed that progenitors from this cluster expressed genes differently from the non-DM controls [50]. In our study, the cell samples comprised of diabetic and non-diabetic hASCs expanded in vitro for three to four passages, showing similar cluster distribution with merely slight differences in cluster constitutions between DM and control cells. Nevertheless, further differential gene expression analysis revealed that ECM organization-related genes were significantly attenuated in diabetic hASCs, and angiogenesis-related genes were differentially altered in the expression level. These findings confirmed the potential functional differences of diabetic and non-diabetic hASCs and highlighted the importance of optimizing specific culture condition for reviving diabetic ASCs.

Corresponding to our in vivo data, some previous studies have shown the therapeutic potential of diabetic ASCs. Sun et al. found decreased expression of VEGF and HGF in murine diabetic ASCs when compared with control ASCs. While these cells were less effective in promoting wound healing compared to non-diabetic ASCs in vivo, the healing effect was superior to the control group treated with PBS [51]. Nambu et al. also showed significantly accelerated wound healing with treatment of atelocollagen matrix containing murine diabetic ASCs in an autologous fashion [52, 53]. These studies and our results suggested that ASCs from individuals with DM can still enhance diabetic wound healing despite impaired cellular function. Thereafter, optimizing in vitro culture conditions to revive diabetic ASCs are still desired for their clinical application for wound healing.

Our previous study found higher ROS generation in hASCs under HG culture condition [27]. The current study revealed a similar finding in dbASCs cultured in HG medium. The stress-induced cell senescence and apoptosis caused by HG environment have been attributed to mitochondrial dysfunction, mainly driven by the overproduction of ROS during carbohydrate metabolism [54–56]. Although a low to moderate level of intracellular ROS can stimulate proliferation, migration, and stemness of ASCs [55, 57], excessive ROS production leads to subsequent inflammation and stress signaling cascades through inhibition of phosphoinositide-3-kinase-AKT pathway or activation of mitogen-activated protein kinase (MAPK) pathway [58, 59]. Hence, cell pretreatment methods targeting at ROS manipulation have been attempted to regain the impaired function of diabetic ASCs. For example, ascorbic acid has been employed in various cell culture medium recipes for its ability to suppress the generation of ROS induced by HG condition [60, 61]. In the current study, culturing diabetic ASCs in LG condition decreased the intracellular ROS level, which may consequently contribute to the better cellular function and wound healing effects.

In this study, significant upregulation of HGF was noted in dbASCs in LG culture condition, which was confirmed by a significantly higher concentration of HGF in CM-dbLG relative to CM-dbHG. Secretion of HGF, along with other angiogenic growth factors, such as VEGF and FGF2, is credited for mediating the wound healing and angiogenesis effect of ASCs [62–64]. HGF also acts as a mitogen and stimulates migration as well as proliferation of various cell types, including endothelial and epithelial cells, via the HGF/c-Met signaling pathway [62, 65, 66]. Particularly, suppression of HGF has been demonstrated to debilitate the therapeutic effect of ASCs in a mouse hindlimb ischemia model [67]. Therefore, enhanced expression of HGF in LG-cultured dbASCs probably contributed to the enhanced proliferation and tubulogenesis of HUVECs in vitro, as well as better healing quality of diabetic wounds in vivo.

Regarding the cell source, allogeneic MSCs are considered as potential sources of cell products to promote diabetic wound healing [5, 68]. Despite being immune privileged or immune evasive, allogeneic MSCs may still stimulate innate immune response in recipients, potentially leading to an inflammatory response and suboptimal wound healing performance [21, 23]. Therefore, the application of autologous ASCs for chronic wounds in DM patients is still a potentially valuable treatment option. For instance, Chang et al. have demonstrated a better wound healing result when applying autologous rather than allogeneic ASCs in a rat burn model [22]. Collectively, the current study shows that the impaired wound healing effect of diabetic ASCs can be partially restored under a LG culture condition in vitro, which is of value for autologous ASC expansion and transplantation in diabetic patients.

## Conclusions

In this study, we compared the characteristics of diabetic ASCs cultured in medium with high- and low-glucose concentration. Enhanced cell proliferation, migration and HGF secretion were found in those expanded in a condition with low-glucose level. This phenomenon may be mediated through the decreased ROS generation of diabetic ASCs in LG culture condition. The in vivo data further revealed enhanced wound healing with more angiogenesis and collagenous tissue deposition in the wound tissue when LG-cultured dbASCs were applied. The improved capability of wound healing effects may be attributed to the enhanced cellular retention of LG-cultured dbASCs relative to their HG-cultured counterparts. This finding encourages the use of low-glucose culture medium for expanding diabetic ASCs in vitro to promote wound healing, which may exhibit important clinical implication for autologous ASC therapy to treat other DM-related complications as well.

### Supplementary Information

**Additional file 1: Table S1.** Primer sequences of mouse genes used for the real-time qPCR analysis.

## Data Availability

The raw single-cell RNA-seq data were deposited in the Gene Expression Omnibus (GEO) under accession number GSE229387. The data can be accessed through the following link- https://www.ncbi.nlm.nih.gov/geo/query/acc.cgi?acc=GSE229387.

## Abbreviations

ASC: Adipose-derived stem cell
MSC: Mesenchymal stem cell
DM: Diabetes mellitus
ECM: Extracellular matrix
VEGF: Vascular endothelial growth factor
FGF2: Fibroblast growth factor-2
HGF: Hepatocyte growth factor
GAPDH: Glyceraldehyde 3-phosphate dehydrogenase
ELISA: Enzyme-linked immunosorbent assay
HUVEC: Human umbilical vein endothelial cell
EGM2: Endothelial growth medium 2
EBM2: Endothelial basal medium 2
ROS: Reactive oxygen species
PBS: Phosphate-buffered saline
FBS: Fetal bovine serum
LDH: Lactate dehydrogenase
SA-β-Gal: Senescence-associated β-galactosidase
BrdU: 5-bromo-2'-deoxyuridine
qPCR: Quantitative polymerase chain reaction
CM: Conditioned medium
t-SNE: t-distributed stochastic neighbor embedding
