# Supplementary material for: Low-glucose culture environment can enhance the wound healing capability of diabetic adipose-derived stem cells
Source: Stem Cell Res Ther. 2023 Sep 4;14:236. doi: 10.1186/s13287-023-03478-2 (PMC10478288; doi:10.1186/s13287-023-03478-2)
Supplement: Supplementary file 1 — Additional file 1: Table S1. Primer sequences of mouse genes used for the real-time qPCR analysis. [file 13287_2023_3478_MOESM1_ESM.docx]

**Additional file 1**

**Table S1.** Primer sequences of mouse genes used for the real-time qPCR analysis

| Target genes |  | Primer sequences |
| --- | --- | --- |
| *FGF2* | Forward | 5’-CACCAGGCCACTTCAAGGA-3’ |
|  | Reverse | 5’-GATGGATGCGCAGGAAGAA-3’ |
| *VEGF* | Forward | 5’-AACGATGAAGCCCTGGAGTG-3’ |
|  | Reverse | 5’-GACAAACAAATGCTTTCTCCG-3’ |
| *HGF* | Forward | 5’-CACCCCTTGGGAGTATTGTG-3’ |
|  | Reverse | 5’-GGGACATCAGTCTCATTCAC-3’ |
| *GAPDH* | Forward | 5’-CAAGGCTGAGAACGGGAAGC-3’ |
|  | Reverse | 5’-AGGGGGCAGAGATGATGACC-3’ |
